# Beyond Where to How: A Machine Learning Approach for Sensing Mobility Contexts Using Smartphone Sensors ^†^

**DOI:** 10.3390/s150509962

**Published:** 2015-04-28

**Authors:** Robert E. Guinness

**Affiliations:** Finnish Geospatial Research Institute, Geodeetinrinne 2, FI-02430 Masala, Finland; E-Mail: robert.guinness@nls.fi; Tel.: +358-50-362-2796

**Keywords:** context awareness, smartphone sensors, machine learning, classification, mobility context, supervised learning

## Abstract

This paper presents the results of research on the use of smartphone sensors (namely, GPS and accelerometers), geospatial information (points of interest, such as bus stops and train stations) and machine learning (ML) to sense mobility contexts. Our goal is to develop techniques to continuously and automatically detect a smartphone user's mobility activities, including walking, running, driving and using a bus or train, in real-time or near-real-time (<5 s). We investigated a wide range of supervised learning techniques for classification, including decision trees (DT), support vector machines (SVM), naive Bayes classifiers (NB), Bayesian networks (BN), logistic regression (LR), artificial neural networks (ANN) and several instance-based classifiers (KStar, LWLand IBk). Applying ten-fold cross-validation, the best performers in terms of correct classification rate (*i.e.*, recall) were DT (96.5%), BN (90.9%), LWL (95.5%) and KStar (95.6%). In particular, the DT-algorithm *RandomForest* exhibited the best overall performance. After a feature selection process for a subset of algorithms, the performance was improved slightly. Furthermore, after tuning the parameters of RandomForest, performance improved to above 97.5%. Lastly, we measured the computational complexity of the classifiers, in terms of central processing unit (CPU) time needed for classification, to provide a rough comparison between the algorithms in terms of battery usage requirements. As a result, the classifiers can be ranked from lowest to highest complexity (*i.e.*, computational cost) as follows: SVM, ANN, LR, BN, DT, NB, IBk, LWL and KStar. The instance-based classifiers take considerably more computational time than the non-instance-based classifiers, whereas the slowest non-instance-based classifier (NB) required about five-times the amount of CPU time as the fastest classifier (SVM). The above results suggest that DT algorithms are excellent candidates for detecting mobility contexts in smartphones, both in terms of performance and computational complexity.

## 1. Introduction

Devices equipped with Global Navigation Satellite System (GNSS) receivers are approaching ubiquity with the inclusion of GPS receivers (or GPS + GLONASS receivers) in nearly all modern smartphones and increasingly in other highly mobile devices, such as tablets and so-called “smart watches”. Although the GNSS receivers in consumer electronic devices tend to provide a less accurate positioning result than professional-grade receivers, the quality and reliability is adequate for many navigation and user tracking applications, as well as a growing number of location-based services. Certain issues continue to challenge researchers, such as obtaining reliable positioning in indoor and urban canyon environments, but it can be argued from some perspectives that the question of “where” has largely been solved by the wide availability of low-cost, low-power-consuming GNSS receivers.

In addition, nearly all smartphones are equipped with a variety of other sensors, such as accelerometers, gyroscopes and digital compasses, which could be used to understand the nature of the user's movement. This enables the smartphone to be aware of not only where the user is but how he or she is moving. This provides additional contextual information, which may be useful for a variety of different applications. A few examples envisioned within this study include detection that: “the user is riding a bus”, “the user is driving a car”, “the user is walking”, *etc.* We use the term mobility context to refer to contextual information related to the user's mode of mobility.

The question of how a person is moving is related to the long-sought goal in mobile and ubiquitous computing of creating context awareness. It is certainly not the only relevant question in context awareness, but a solution that could reliably answer it would provide significant value in terms of enabling context-aware applications. For a recent review of context-aware computing, see [1].

There are many potential applications where mobility context could be used: an application that automatically searches bus or train timetables as the user walks towards a stop or station and accordingly plans his or her route, an application that tracks a user's carbon footprint as a result of using a car or other means of transportation, functionality in a smartphone to automatically respond to incoming phone calls with a message “Sorry, I'm driving…” whenever the user is driving or an application to track a user's physical activity (walking, running, *etc.*) for personal health monitoring. In addition, mobility context can be used in the navigation subsystem of a smartphone in order to improve the navigation solution. For example, when the smartphone detects that the user is walking, it could apply an optimized Kalman filter to fuse measurements from the various sensors and account for shortcomings of the GNSS receiver (e.g., in an urban canyon environment). The mobile computing literature provides many other examples of application concepts where mobility context plays a strong role (e.g., [2]).

Despite the great potential for applications of mobility context, a reliable solution to provide this type of information is not yet available commercially, although several companies have attempted such offerings. This depends, of course, on one's definition of reliable, but a review of user feedback to these commercial offerings suggests that users are not yet satisfied with the accuracy of existing solutions. In addition, a frequent complaint is regarding the increased energy usage of these solutions (*i.e.*, quickly draining the smartphone's battery). This suggests that the designers should take into account the computational complexity of the algorithm that determines the mobility context.

Several researchers have performed relevant studies on mobility context recognition. For example, [3] used a mobile phone with a GPS receiver and accelerometer to determine transportation modes of users, achieving 93.6% classification accuracy with the use of discrete hidden Markov models. They did not, however, distinguish between different types of motorized transport (e.g., car, bus, train, *etc.*), but grouped all of these into a single class. Bancroft *et al.* [4] used a foot-mounted device (with GPS receiver and inertial measurement unit) to determine different motion-related activities, such as walking, running, biking and moving in a vehicle, reporting less than 1% classification error during 99% of measurements. This study focused on applications for military personnel and first responders, where the use of specially-designed foot-mounted devices is feasible. Although foot-mounted accelerometers are gaining in popularity (e.g., Nike + iPod sensor), they are not yet ubiquitous in usage. Thus, solutions that rely on foot-mounted devices are of limited applicability in the current consumer market.

Accordingly, our long-term goal is to develop a solution that detects a wide range of mobility contexts (walking, running, biking, car, bus, train) using only devices that are truly ubiquitous in today's environment. Smartphones are an obvious choice, due to the rich set of sensors that they possess and their widespread use among the general public. According to market research firm Strategy Analytics, the number of smartphones in use worldwide surpassed one billion in the third quarter of 2012, and they estimate that this figure will double by 2015 [5]. Thus, if a solution for the mobility context can be developed that relies only on a smartphone, its potential impact is enormous.

This paper is an extension of a previously published conference paper [6], which was, to the authors' best knowledge, the first study to combine the use of GPS, inertial sensors (e.g., accelerometers), information from geographic information systems (GIS) and ML techniques in order to detect mobility contexts. Several studies have used GPS, GIS and accelerometers with the aim of understanding transport-related physical activity, and [7] provides a relatively recent review of this literature. A few relevant studies have been published in more recent years. For example, [8] used GPS, GIS and accelerometers to assess travel behaviors. No ML techniques, however, were used to classify the behavior into different mobility contexts. Furthermore, the study was not implemented using smartphone-based hardware. Elhoushi *et al.* [9] studied the use of GNSS and micro-electro-mechanical system (MEMS) sensors, together with ML techniques, to detect the mobility context. The authors report overall accuracy of nearly 95%; however, they do not specify which ML algorithm was used for classification. Other studies have combined GPS and accelerometer data with GIS information, but for other purposes, such as identifying the areas where children exerted the most physical activity [10]. What is consistently lacking in many existing studies involving GPS, accelerometer and GIS information is a systematic use and evaluation of available ML techniques for classification purposes, which is surprising given the success of ML techniques in many other disciplines.

There are several studies utilizing ML and accelerometer data. Jin *et al.* [11] detected several motion states using an armband-mounted accelerometer and a “fuzzy inference system”, which can marginally be considered an ML technique. Other studies that used both accelerometer data and standard ML techniques to determine mobility or activity-related contexts include [12–16]. They are all lacking, however, in the use of GPS or GIS. There has been one study that used ML, GPS and GIS, but not accelerometers [17]. In a later work by Stenneth, however, accelerometers were also used [18]. From certain aspects, this is the most similar to our work compared to all other existing studies.

The main goal of this present study is to determine if a machine learning technique exists that, given information provided by GPS, inertial sensors and GIS, can produce a classifier having both high performance (in terms of recall rate) and low computational complexity. If this can be achieved, then it offers strong support for the feasibility of detecting the mobility context using smartphones.

This paper is organized as follows. First, we describe in greater detail the overall methodology used for creating a mobility context classifier. Next, we depict the experiments that we conducted in order to collect data from various mobility contexts using a smartphone, including a description of the application developed for this purpose. Thirdly, we discuss the specific procedures used to process and analyze the collected data and present the results of the experiment. Finally, we draw some conclusions and discuss our future work in this research topic.

## 2. Methodology

Since our goal is to detect mobility contexts, such as “user is walking” and “user is riding a bus”, the main task is one of classification. One approach would be to use unsupervised learning techniques, such as clustering, in order to organize the data into groups (*i.e.*, classes) of high similarity without specifying the groups *a priori*. In our case, however, we desire to obtain groups that have a clear real-world interpretation, so it is more appropriate to define them according to our natural intuitions about the mobility context. Furthermore, we would like some measure of “performance” of our resulting classifier that is easy to interpret. An obvious choice is the rate at which the classifier produces the “correct” classification of the user's motion, *i.e.*, the recall rate. Thus, it is necessary to manually label the data instances with the correct class, leading to the methods of supervised learning.

**Figure 1:**
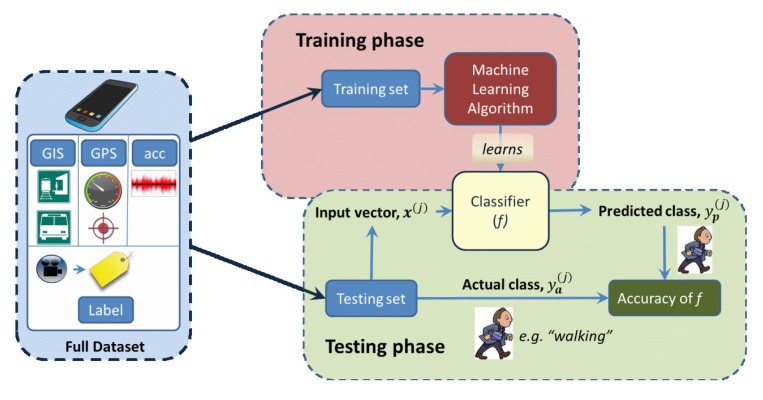
Overview of the supervised learning process, as used in this study.

The basic process used in this study is summarized in Figure 1, which follows the standard supervised learning approach. In the training phase, a function is generated that maps each of the input vectors to a target class:

(1) f:x→y

where *x* is the input vector and *y* ∈ *Y* = {*y*_1_,*y*_2_, …*y_m_*}, *i.e.*, the set of all possible classes. The exact form of this function depends on the ML algorithm being used. In some cases, the algorithm produces a probabilistic model of the class distributions, given the input vector, *i.e.*, *p*(*y*|*x*). The function is made deterministic by selecting the most likely class according to:

(2) $$y=\underset{y\in Y}{\arg\max}p\left(y|\text{x}\right)$$

Finally, after the function is learned, its performance is measured during the testing phase (using the testing set), where the values of *y* output from the function *f* are compared to the labeled values (*i.e.*, the “correct” classes). Note that the same function *f* is used in both the training and testing phases.

Because the performance will exhibit some variance, due to the finite size of the testing set, this process is repeated and the results averaged. Specifically, we use 10-fold cross-validation.

## 3. Experiment Design and Data Collection

For this study, we implemented a smartphone application for the Android operating system capable of capturing accelerometer and GPS data. This application, called *CommutingContext*, also performed several data processing and information retrieval functionalities, which will be described in further detail below. The application was tested on a Samsung Galaxy Nexus smartphone, which is equipped with a SiRF StarIV GSD4t GPS receiver and Bosch BMA180 three-axis accelerometer [19]. The smartphone was running Android Version 4.1 (“Jelly Bean” release).

The data were collected over the course of one day during summer 2012 using two male test subjects, aged between 30 and 40 years and of good physical health. As this study is of a preliminary nature, no attempts were made to control for or analyze the possible effects of age, gender, height or other characteristics of the test subjects on the collected data. All data were collected in the western Uusimaa region of Finland.

The test subjects were asked to perform various mobility activities, including walking, running, riding a bus, riding a train and driving a car and to do so in the same manner as they naturally would. The only departures from normality were that the test subjects were asked to shake the phone at the start and end of each test run (used as a signal for later calibrating the time system), and they were also asked at various points during the tests to assume a “static” motion state (primarily after or before a “walking” motion state). Lastly, the subjects were instructed to place the phone in their pants pocket, in order to maintain some consistency in the smartphone's placement and orientation.

In total, fifteen test runs were performed, varying in length and mobility contexts. Some details of these test runs are presented in Table 1. Each of these test runs were either video + audio recorded or audio recorded by a dedicated device, operated by an observer. The purpose of this was to allow us to manually “label” the true mobility context as a function of time, providing a reference to measure our classification performance against. About halfway through the experiment, we switched from video + audio recording to audio-only recording, because we noted that this was sufficient for reference purposes and also due to the potential privacy concerns with recording video on public buses or trains.

### 3.1. The CommutingContext Application

**Figure 2:**
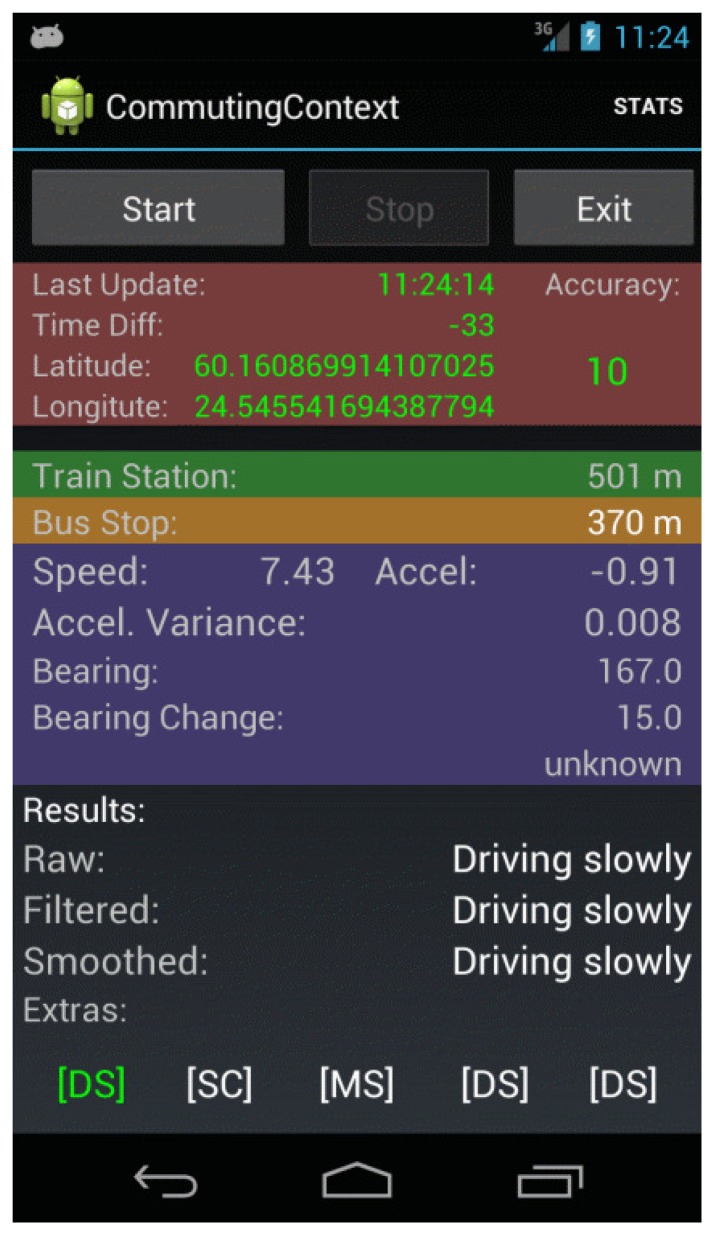
Screenshot of the CommutingContext application.

The main purpose of the CommutingContext application was to collect GPS and accelerometer data and to perform some basic data processing and information retrieval functions. A screenshot of this application is shown in Figure 2.

One of the information retrieval functions that the application performed was to look up train stations and bus stops in the vicinity of the user, using the position provided by the GPS and the smartphone's wireless data connection. Typically Universal Mobile Telecommunications System (UMTS) or Evolved High-Speed Packet Access (HSPA+) connections were available during the experiment. Two separate web-based services were used for these purposes: Google Places API for train stations [20] and the Reittiopas API, provided by the Helsinki Regional Transport Authority [21]. The primary reason for using Google Places API was its global availability. For bus stops, however, the Google Places API was not comprehensive enough for the areas where the experiment was to be performed. The Reittiopas API, on the other hand, is highly comprehensive and accurate, but is limited in scope to the areas serviced by the Helsinki Region Transport (HRT) system (known in Finland as Helsingin seudun liikenne (HSL)).

Both of these web services return an array of station/stop objects for a given set of latitude/longitude coordinates, ordered from nearest to furthest away. The application then uses the Android API's built-in method for calculating the precise distance between the user and the nearest station/stop.

Another important function of the application was to perform data processing operations, especially with respect to the accelerometer data stream. Previous studies [14,22] have shown that an important feature for motion classification is the variance (*σ*^2^) in the norm of the acceleration (|*a*|), defined by the following equations:

(3) $$\left|a\right|=\sqrt{a_{x}^{2}+a_{y}^{2}+a_{z}^{2}}$$

(4) $$\sigma_{t}^{2}=\sigma_{t-1}^{2}+{\bar{\left|a\right|}}_{t-1}^{2}-{\bar{\left|a\right|}}_{t}^{2}+\frac{{\left|a\right|}_{t}^{2}-{\left|a\right|}_{t-N}^{2}}{N}$$

where ${\bar{\left|a\right|}}_{t-1}$ and ${\bar{\left|a\right|}}_{t}$ represent the mean of |*a*| at times *t* − 1 and *t*, respectively, and *N* is the size of the window over which σ^2^ is calculated. Because σ^2^ is calculated recursively, the memory required for its calculation is bound by the window size, *N*. In our case, we used a window size of 50 accelerometer measurements, which corresponds to slightly more than a one-second window. Preliminary tests conducted during application development showed that the resulting acceleration signal produced from this feature was sufficiently responsive to motion changes, such as walking and stopping. Using the norm of acceleration also has the beneficial property that it is invariant under changes in the orientation of the smartphone.

In total, the application recorded in a log file a total of fourteen features. Due to the importance of GPS in this experiment, no data were recorded if there was an outage in the GPS solution. For debugging purposes, a subset of these features was displayed in the user interface (see Figure 2). In addition, the raw accelerometer data were recorded in a second log file for post-processing purposes. One additional feature, which will be described in detail below, was produced from this accelerometer data during post-processing.

## 4. Data Analysis

Now, we describe how the above methodology was implemented in this study. The first task was to manually label the 8498 data instances with the correct mobility context, provided by the reference recordings. A total of seven motion classes were defined, including: static, moving slowly, walking, running, driving a car, riding a bus and riding a train. The distribution of these classes in the dataset is shown in Table 2. Due to the differences between the time systems in the test smartphone and the reference recording device, a “shake” signal was used to perform time calibration. This shaking of the phone, which could be seen clearly in the acceleration data, allowed us to synchronize between the two time systems for labeling purposes. Each mobility context and the associated transitions could be clearly recognized in the video recordings, and the times of the transitions were noted. For the cases where audio recordings were used, transitions were verbally noted by the observer during the experiment; thus, the transition times were noted in a similar manner during post-processing.

In addition, some other simple post-processing steps were performed in MATLAB, such as filling in labels between the mobility transitions and removing extraneous data from the beginning and end of the data files. We also removed data corresponding to circumstances not reflected in any of the classes, for example walking up/down stairs near a train station (these times were noted in the reference recordings). Finally, the data were formatted in MATLAB into a form that could be used in Weka, the software used for the majority of our data analysis. The entire dataset is available via GitHub, see [24].

### 4.1. Selecting Features for Classification

The data contained a total of fifteen features, not including the class label. Four of these features were related to the time system (elapsed time, wall time, Unix time and GPS time). Although some mobility contexts could be correlated with respect to time of day (particularly for specific individuals, whose commuting activities follow some routine), we wish our final solution to perform well independent of such factors. Therefore, we removed these as classification features. Similarly, we removed latitude, longitude and heading as features for consideration, since we would not expect these to be invariant across all users. We considered the remaining eight features, described in Table 3, all to be candidates for use in classification.

As mentioned above, one feature was not computed in real-time during the data collection, but instead was computed during post-processing. This is the “1 HzPeak” feature. As this feature requires a transformation to the frequency domain, we chose not to attempt to compute it in real-time for this research. Although modern smartphone processors are certainly capable of computing such features in real-time, using a fast Fourier transform (FFT) algorithm, for simplicity, we decided to perform this step in post-processing. It was computed by taking an FFT of the variance of the norm of the acceleration (as described in Section 3.1 above), using a sampling frequency of 125 Hz and operating on four-second windows of accelerometer data. After examining the spectrum plots for many segments of the training data, we decided to use as a feature the relative strength of the FFT peak at about 1 Hz. This feature *p_r_* was computed as:

(5) $$p_{r}=\frac{2*p_{0.98Hz}}{p_{0.73Hz}+p_{1.22Hz}}$$

where *p*_0.98_*_Hz_*, *p*_0.73_*_Hz_* and *p*_1.22_*_Hz_* are the peaks at 0.98 Hz, 0.73 Hz and 1.22 Hz, respectively. The feature *p_r_* can be intuitively understood as a signal related to walking, as most people normally walk with a gait around 1 Hz in frequency. This is supported by previous research and also was evident in our data in spectrum plots corresponding to periods of walking.

### 4.2. Algorithm Performance Comparison Using Weka

Weka is an open-source, widely-used software framework for ML and data mining [23]. It was chosen because it supports a large number of ML algorithms, which have been tested and verified using many different reference datasets. It is also suitable for developing new ML algorithms, although all of the algorithms used in this study are standard ones distributed with the software package.

Included in the Weka software package (Version 3.6.8) is the Weka Experiment Environment, which allows one to perform repeated model training using different ML algorithms and employing standard performance testing techniques, such as cross-validation. We used this environment to perform classification on our dataset using a wide range of ML algorithms. Each algorithm was used to train and test a model using 10-fold cross-validation. Because performance varies depending on how the folds are partitioned, this procedure was repeated 10 times for each algorithm, in order to obtain adequate statistics regarding classification accuracy. The full set of algorithms we employed, grouped by type, is listed in Table 4, along with the key performance metric, classification accuracy (also known as recall rate).

In Table 4, we use naming conventions consistent with the Weka software package. These sometimes differ from naming conventions used in the ML literature. For example, J48 is a java implementation of C4.5, which was originally developed by Quinlan (see [25]). Due to space limitations, we will not attempt to describe all of these algorithms in detail, but refer the reader to [26] or the Weka software documentation for details [23]. Several of the algorithms will be described further in the Discussion Section.

Here, we will mention only one algorithm, ZeroR, which provides a baseline for comparing to the other algorithms. This is a trivial algorithm that simply classifies all of the testing instances as the most frequent class in the training set (in our case, the “walking” class). Thus, all other algorithms should perform at least as well as ZeroR (as is the case here).

It is important to note that all of the algorithms included in the Weka software package assume that they will be applied to independent and identically distributed (i.i.d.) data. In fact, in nearly all of the ML literature, it is assumed that the data of interest is i.i.d. In our application domain, however, this is clearly not the case. Time series data from different mobility contexts are expected to be highly correlated in time. This issue is handled in Weka by randomizing the order of the data instances, which obscures the time dependence. See the Discussion section for further implications of this issue.

### 4.3. Feature Analysis

Because ML algorithms can sometimes suffer poor performance due to the “curse of dimensionality”, we next endeavored to determine if better performance can be achieved by using a subset of the seven features described above. Feature analysis is also of particular importance in this research domain because different features have different costs, in terms of the amount of energy required to produce them. Thus, feature analysis provides a means of multi-objective optimization, e.g., when the goal is to achieve adequate performance while minimizing energy requirements.

In order to perform feature analysis, we performed a combination of greedy forward feature selection (GFFS) and greedy backward feature selection (GBFS). We first re-ran the analysis for five algorithms (chosen because of their good performance and diversity) using each feature individually (*i.e.*, in each analysis, only a single feature was utilized). The results, presented in the top section of Table 5, indicate that speed, acceleration variance (accelVariance), distance to train station (trainDistance), speed change (speedChange) and distance to bus stop (busDistance) are among the most important features individually. In a similar fashion, we re-ran the analysis using all of the features minus a single feature (varying the missing feature each time). These results, shown in the second sub-section of Table 5, show that, for most of the algorithms and features, the classifiers do not degrade significantly when a single feature is removed. In fact, for the case of RandomForest with speedChange removed, the resulting classifier performed slightly better, and when headingChange was removed, there was a negligible change in the performance. Furthermore, note that speedChange, although among the top individually performing features, actually degrades the performance when a larger set of features is used.

Next, we re-ran the five classifiers using only time-domain features and removing a single feature among the seven remaining features. The reason we were interested in these results is because, in some applications, it may be too costly in terms of energy to generate frequency-domain features. We wanted to better understand the performance without the 1 HzPeak feature. To see the effects of removing individual time-domain features, the results in the third sub-section of Table 5 can be compared to the second to last row in the second sub-section, where all time-domain features were used.

Lastly, we re-ran the analysis using various small subsets of the features. A sampling of these subsets is shown in the last sub-section of Table 5. None of these subsets were the best overall performer, but one subset ({av, s, t, b, acc, 1hp}) was nearly as good and surpassed nearly all of the classifiers that used all available features. Note also the subset containing all but the GIS-related features (*i.e.*, {av, s, h, acc, sc, hc, 1hp}) contains all of the features, except the two GIS-related ones. Its performance was about 3.4% less than when using all features. This highlights the usefulness of GIS in this domain. This difference would most likely have been more significant if the dataset had consisted of more instances of “riding a bus” and “riding a train” (see Table 2).

Although the results in Table 5 do not represent an exhaustive feature selection procedure, they offer some support that, with the possible exceptions of speedChange and headingChange, the features used in this study are useful for classification and have minimal redundancy.

### 4.4. Parameter Tuning

Most ML algorithms specify one or more parameters that can be tuned in order to optimize performance or other characteristics, such as the tendency to overfit the training data. The Weka platform supplies the user with default parameters for each algorithm, usually based on some rule of thumb or generally accepted set of values. Not all algorithms, however, have a set of universally applicable parameter settings and should ideally be tuned according to training data. We selected the RandomForest algorithm for further parameter tuning, because it performed the best in most of the above evaluations. For parameter tuning, we used as our input data the feature set containing all features, except speedChange. This choice was made because the feature analysis presented above showed that this was the best set of features using the default parameter settings for RandomForest.

In the Weka implementation of RandomForest, there are three parameters that are user-defined: (1) the max depth of the decision trees; (2) the number of attributes (*i.e.*, features) used for random selection during the learning process; and (3) the number of trees grown. Early analysis showed that the recall rate was not very sensitive to the first parameter. We tested the performance at increasing values for this parameter and found that somewhere in the range of 20 to 30, the performance leveled off and remained constant for higher values. It is also possible to set this parameter such that no constraint is placed on tree depth, and this is the setting we used in further analysis.

The default value in Weka for the second parameter (number of attributes used in random selection), which we call *F*, is ⌊*log*_2_*M* + 1⌋, where *M* is the total number of attributes available. For our dataset, this parameter would default to *F* = 3. We wanted to determine if this was indeed the best setting for this parameter or if better performance could be achieved with another value. Regarding the last parameter, the number of trees grown (denoted by *K*), it is generally accepted that the generalization error converges to a minimum as *K* increases. We wanted to see at which value an increase in *K* provides negligible performance benefit, since increasing this also increases the complexity of classification.

**Figure 3:**
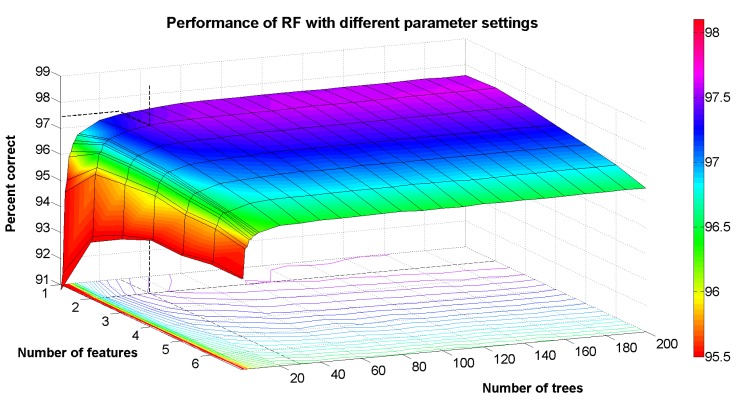
Performance of Random Forest as a function of parameter settings.

In order to avoid the problem of overfitting from the parameter tuning process, we created a hold-out test set of 10% from the original dataset using random sampling without replacement. As a result, the class distribution of the hold-out test set is similar, but not identical to the class distribution of the entire dataset. With the remaining 90% of data, we performed training and evaluation of the RandomForest classifier using 10-fold cross-validation, while varying the parameters *F* and *K* using a grid search approach. The results are shown in Figure 3. From the contour lines, we can see that for a small choice of *K* (<10), there is no clear best choice of *F*. However, for *K* > 10, the best choice of *F* is clearly two. This can be seen from the contour lines, because for all other values of *K*, a greater number of trees is needed to achieve the same level of performance as for *F* = 2. We have highlighted one particular point on this curve, where *F* = 2 and *K* = 30. The performance at this point is about 97.444%, which, compared to the point *F* = 2 and *K* = 200, is only 0.2% worse in relative terms. Finally, we set *F* = 2 and re-trained the RandomForest classifier using the full 90% training data, but varying the value of *K*, and evaluated the resulting performance using the 10% hold-out test set. The resulting performance was 97.291% correctly classified samples. The purpose of the analysis using the hold-out test set is to provide an estimate of how the classifier will perform on new, unseen data; however, to produce an estimate of good confidence, a large amount of labeled data is needed. Given that this hold-out set only contains 849 data samples and, for some classes, only a few tens of samples, the main value of this estimate is that it lends support to the fact that the parameter tuning process did not introduce significant overfitting. Taking together, the parameter tuning results and the hold-out set results provide good evidence that a classification performance of around 97% can be achieved, although we caution the reader that results from two test subjects moving about in one geographic region do not necessarily generalize to a larger, global population of smartphone users.

Lastly, the results from the parameter tuning analysis suggest that for the types of applications described in this paper, the choice of *K* could be a user-selectable parameter (e.g., 10 ≤ *K* ≤ 30), since higher values will improve the performance, but also increase the computational complexity of the classifier. Clearly, however, the results in Figure 3 show that increasing *K* to values above about 30 will only provide minuscule improvements in performance, and it is a case where the law of diminishing returns holds strong.

### 4.5. Computational Comparison of Algorithms

In order for a classifier to be feasibly implemented in a smartphone, its underlying classification algorithm must be relatively simple; otherwise, classification cannot be performed in real-time or would require an unreasonable amount of resources, draining the limited battery power of a smartphone. For this reason, we next analyzed the computational complexity of the classifiers. We expect there to be a direct linear relationship between computational complexity and battery usage, although this must be confirmed in future studies (see [27]).

First, an important distinction must be made regarding the operations that we are analyzing. As described above, ML algorithms necessarily involve two steps: model training and testing (in our case, classification). In this analysis, the only portion that we are interested in is testing, because this is what would be implemented in a smartphone in order to perform real-time classification. Model training, on the other hand, is likely to be performed on a PC (or server) and the resulting models later transferred to a smartphone. For most ML algorithms, model training requires vastly more computations than testing/classification.

Measuring the relative complexity of the classification algorithms is straightforward, because Weka can measure and save the CPU time used during the testing portions of each analysis run. Although these times are likely to vary with the system and platform being used, they are suitable for purposes of comparing the different algorithms. These tests were performed on a PC with an Intel Core i5-2450M processor with a 2.5-GHz clock speed. The results are presented in Table 6.

The times represent the average amount of CPU time used to classify 1/10th of the samples in the dataset (about 850 instances). To aid the reader in understanding the presented values, consider a system that classifies the user's mobility context at a rate of 1 Hz. If RandomForest were used, then the classification part of the computation would require about 10 μs of CPU time each second, whereas the classifier based on IBkwould require nearly 1 ms of CPU time each second. While both may be feasible in principle to operate at a rate of 1 Hz, clearly the IBk classifier would consume significantly more energy than the RandomForest classifier. Of course, the presented values were measured on a PC rather than a smartphone, so the actual values in a smartphone-based implementation will differ from those presented. The ratios between different classifiers in terms of CPU time, however, should remain relatively consistent over similar implementations on different processors.

The CPU time measurement procedure was repeated 100 times (10 repetitions of 10-fold cross-validation). Note that the standard deviation of many of these measurement times is high compared to the actual times, especially for the faster algorithms. This is due to limitations in the precision of the CPU time measurement provided by Weka. In the future, we aim to measure the CPU time for classification using larger datasets and directly on various smartphone CPUs. The presented results suffer from our current software limitations, but the main value at this stage in our result is to provide a comparison between different algorithms.

From the above results, we can conclude that most decision tree-based algorithms are very fast compared to other algorithms. SMOand MultiLayerPerceptronare also very fast algorithms for the testing portion. Instance-based classifiers, on the other hand, are extremely slow for testing. This will be discussed in further detail in the Discussion Section. Although the absolute speeds will certainly vary under different processors and architectures, we have no reason to believe that the relative speed of these different classifiers will differ significantly from one CPU to another. Nonetheless, this claim should be confirmed in future work.

It is also true that future implementations of, for example, instance-based classifiers could be parallelized and optimized for graphics processing units (GPUs), which are common in many modern smartphones. Recent research results have shown a ten- to twenty-fold speedup in classification utilizing GPUs and parallel versions of the k-nearest neighbors (kNN) algorithm [28]. A detailed consideration of parallelization, however, is beyond the scope of the present study.

### 4.6. Error Analysis

Lastly, we measured the types of errors that occurred for the best classifier, *i.e.*, RandomForest, using all features, except speedCahnge. Classification errors are typically presented in the form of a confusion matrix, where the true class labels and predicted labels comprise the rows and columns of the matrix, respectively. This result is presented in Table 7 below, where the bold values show the correctly predicted class label and the non-bold values show the various classification errors.

Here, we see that the most common errors include the following incorrect classifications: “moving slowly” as “static”, “running” as “walking”, “static” as “walking”, “moving slowly” as “walking” and “driving car” as “riding bus”. Note that the percentages are out of all instances within the respective class, not out of the whole dataset. The accuracy/error percentages by class give a notion of how the algorithm will perform when the smartphone is engaged in a particular mobility context. For example, “walking” is detected with the highest accuracy, whereas “moving slowly” is detected with the least accuracy, in terms of the correct classification rate.

**Figure 4:**
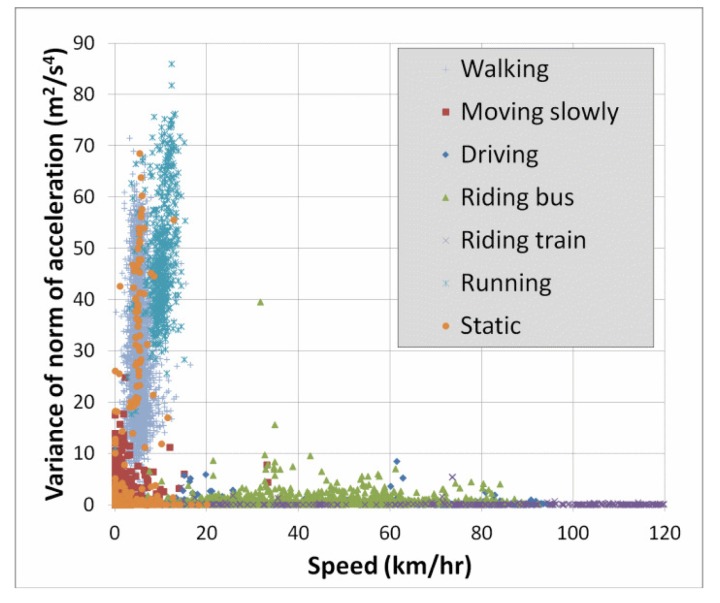
Plot of accelVariance vs. speed for all mobility classes.

**Figure 5:**
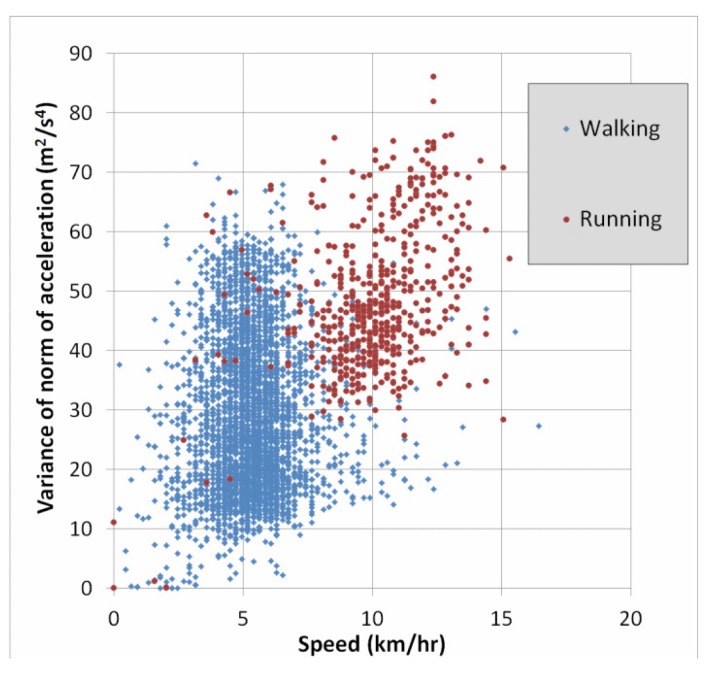
Same plot for only “walking” and “running” classes.

Lastly, a simple way to obtain a better understanding of the cause of errors is to use two-dimensional plots of the features. Since we have seen above that speed and accelVariance are among the most important features, we created plots to see the distribution of the classes with respect to these two variables. Figure 4 below shows such a plot for the entire dataset. For greater clarity, Figure 5 shows only the classes “walking” and “running”. Recall from Table 7 that 6.2% of the “running” instances were confused with the “walking” class. This is not surprising in light of Figure 5, and we will discuss this error type further in the section below.

## 5. Discussion

The main goal of this study was to determine if an ML algorithm exists that can produce a classifier having both high performance (in terms of class prediction rate) and low computational complexity. The results show that several existing ML algorithms achieved performance above 95% accuracy, but the only ones that also have relatively low computational complexity (for classification) are decision-tree algorithms. In particular, the RandomForest and NBTree algorithms performed well. RandomForest, however, surpassed NBTree, both in terms of performance and complexity.

RandomForest is, in fact, an ensemble classifier, meaning it uses multiple classifiers, and the final classification is performed by “voting” among the classifiers. For an overview of the method of random forests, see [29]. Specifically, the Weka implementation is similar to the algorithm referred to as “Forest-RI” in [29]. RandomForest creates an ensemble of such trees using randomly-selected attributes.

There is one difference between Forest-RI and the Weka implementation of RandomForest. Weka uses REPTreeas the base classifier; The name of this algorithm comes from “reduced-error pruning”, which means the decision tree is iteratively “pruned” (*i.e.*, decision nodes are removed) and then tested to see the effect on its performance [30]. Breiman's algorithm uses unpruned trees.

As mentioned above, many of the algorithms used in this study contain one or more parameters that can affect the classification performance. In all cases, except one, we used the default parameters provided by Weka and did not attempt to optimize the parameter settings for performance. The exception is for IBk, which is a classifier based on the kNN algorithm. We tried several values (1, 5, 10, 100) for the k parameter and report in this paper only the best performer (k = 5). Further attempts at parameter optimization of ML algorithms would likely require more labeled data, and as such, it is not emphasized in this study.

Next, we note that two instance-based classifiers also performed well (KStar and LWL) in terms of accuracy. In terms of computational complexity, however, these two algorithms were the worst performers. This is due to the fact that instance-based classifiers essentially work by memorizing the training data. During testing, the algorithms perform classification by searching through the training instances to find the closest match. They are sometimes known as “lazy learners” because they do not make any inferences until the testing phase. As such, they are not particularly suitable for smartphone-based processing, unless a very efficient look-up scheme could be devised.

In general, the models produced by decision-tree algorithms are intrinsically simple, because they consist of simply a set of if-then-else statements, which can easily be implemented in any programming language. The Weka API even has the ability to output a java source representation of the decision trees that it builds. Of course, the size of the tree (*i.e.*, number of nodes) will affect its computational complexity. Our results show, however, that even an ensemble of decision-trees, like RandomForest, is not significantly more complex than, for example, Bayesian techniques or logistic regression. Although the analysis of computational complexity was performed on a PC, the result in relative terms should be similar if all of the classifiers were implemented on a smartphone. Our plan to demonstrate this will be discussed in the section on Future Work.

As mentioned above, all the algorithms available in Weka make assumptions that the data will be i.i.d., which is not strictly the case with this dataset. This does not prevent the algorithms from being used with time series data, but it does mean that the algorithms cannot take advantage of the time dependence existing in the dataset [31].

One simple way to exploit the inherent time dependence would be to consider at each epoch the latest *n*-outputs of the base classifier (e.g., RandomForest) and then produce an ensemble classifier that chooses the majority from the *n* individual outputs. This would in effect “smooth” the classifier results in the same way that a low-pass filter smooths a noisy signal. The disadvantage of this approach is that the resulting ensemble classifier would not react as quickly to changes in the mobility context, since it would rely on a memory of recent context states.

In practice, it is rather challenging to perform this kind of analysis using the techniques of machine learning and the available software tools, although it could be achieved if explicitly planned during the data collection or built into the analysis software. In general, machine learning techniques call for randomized sampling from the labeled data to segregate the data into training and test data, as described above. Since the smoothing technique described above requires consecutive data samples, one would either need to have a method of randomly selecting many small sets of *n* consecutive data samples to segregate the data into training and test sets or one would need to break the violationof random sampling altogether. In the case of the latter, it would be very important to ensure that the data collection is done in a way that some small time series of data from within the whole dataset is representative of the whole dataset, especially in terms of class distribution. This is perhaps impractical, however, and we plan to implement the former technique in our future research.

There exist some other statistical models that utilize time dependence more deeply, such as hidden Markov models (HMMs) or conditional random fields (CRFs), which could provide better results than the ML algorithms examined in this paper. Use of these models for detecting mobility context will be another subject of future research.

Finally, we discuss some potential sources of the classification errors in our results. One likely source is due to error in the speed measurements provided by GPS. For example, we observed that about 27% of the data instances with the “static” class label have a non-zero speed measurement. In fact, more than 17% have a speed measurement >2 km/h. This level of error in speed measurements is probably largely the reason for confusion between the “static”, “moving slowly” and “walking” classes. We attempted to augment the GPS speed measurements using the acceleration data, but due to the large noise levels, this proved unsuccessful. What is clear, however, is that if the error of speed measurements at low speeds could be improved, either by next-generation GNSS receivers, GNSS-IMU integration or some other means, then the classification results will also improve.

One of the most frequent classification errors was when “running” was confused with “walking”. As discussed in the section above, this is due to a significant overlap between these two mobility classes in terms of the features speed and accelVariance. This is perhaps not surprising, because when the user is jogging slowly, the speed is very similar to that of fast walking. The average speed for all walking instances is about 5.34 km/h with nearly 8% of instances having speeds greater than 7 km/h. This can be compared to running, where the average speed is 10.1 km/h, and more than 6% of speed measurements were less than 7 km/h.

In Figure 5, there is an even greater overlap in terms of accelVariance. It is possible that other features of the accelerometer may be able to better distinguish between these two classes, for example the 1 HzPeak feature. Another possibility would be to collect data from additional inertial sensors, namely MEMS gyroscopes, which are available in many smartphones.

## 6. Conclusions

This research demonstrated that, by combining data from GPS, accelerometers and GIS with existing ML algorithms, one can build a highly-performing classifier for detecting mobility contexts of smartphone users. Results from evaluating the performance of various ML algorithms using our full feature set ranged from 80.2% to 96.5% in terms of the correct classification rate. Results from measuring the computational complexity of the classification algorithms suggested that many of these classifiers can feasibly be implemented in a smartphone, although future research must verify these preliminary results.

The only part of the process that would likely need to be implemented separate from the smartphone is the training (model learning) phase, which can be done using a standard PC with the resulting model then being transferred to a smartphone. Since training is performed off-line in supervised ML, it is not required that it be implemented in a smartphone.

Furthermore, our analysis showed that decision-tree-based algorithms and, in particular, the RandomForest algorithm are ideally suited for this type of application. The best performance was achieved when this algorithm was used with six of our seven features (*i.e.*, all features, except speedChange), which resulted in an average accuracy of 97.7%. Although RandomForest is not the least computationally complex of all of the algorithms analyzed, it is feasible to implement on modern smartphones. This conclusion is supported by the fact that it required on average less than 8 ms of CPU time to classify 850 instances, using a PC.

Finally, the most common classification errors were confusion between the “static”, “moving slowly” and “walking” classes, as well as incorrect classification of “running” as “walking” and “driving car” as “riding bus”. In terms of the features used in this study, these are the classes that are most challenging to correctly detect. All mobility classes were correctly classified more than 90% of the time with the exception of “moving slowly”. For this class, correct classification was only achieved 87.9% of the time with nearly 8% of the error going to the “static” class and nearly 3% going to the “walking class”. This is not surprising, given that “moving slowly” was defined somewhat as a bridge between these two classes.

## 7. Future Work

We hope that this work will serve as a baseline for future efforts for detecting mobility contexts. We examined only the ML algorithms included in the Weka software package, so future work will include extending our analysis to other existing ML algorithms, such as those based on hidden Markov models and conditional random fields. Furthermore, we have begun preliminary work on a novel algorithm that combines aspects of decision trees and hidden Markov models, which we plan to develop further.

In addition, we plan to implement several of the algorithms considered in this paper on the Android OS and to measure the CPU usage on several different smartphones, in order to more definitively demonstrate the feasibility of a purely smartphone-based solution.

We also plan to investigate other features that could be used as features for classification, including other frequency domain features, as well as from additional sensors, such as gyroscopes. With regards to gyroscopes, one possibility for the future is to use gyroscope data to reduce the error of the heading measurements, which may result in the headingChange feature being of greater value in future studies. It is also possible that other GIS type information could be used as features for classification. For example, OpenStreetMap offers a web service that allows retrieval of road segments or other “nodes” from its database [32]. These could be very useful for improving the detection of mobility contexts.

Lastly, it is clear that more mobility context data should be collected, including data from a wider range of test subjects, more diverse environments and additional mobility contexts (e.g., cycling). It should not be taken for granted that the performance results achieved from two test subjects will generalize to a larger, more diverse population. Another interesting topic of study would be to investigate the effects of placing the smartphone in different locations other than the user's pocket, for example in a backpack or handbag.

## Figures and Tables

**Table 1:** Test runs comprising the dataset. W = walking; R = running; S = static; MS = moving slowly; RT = riding train; RB = riding bus; D = driving.

**Test Subject**	**No. of Data Instances**	**Time Length**	**Motion Contexts**
Subject 1	582	10:03	W, S
Subject 1	292	11:32	W, S
Subject 2	364	6:21	W, S
Subject 2	636	15:09	W, S, MS, RT
Subject 2	269	10:48	W, S
Subject 2	654	11:08	W, S, RT
Subject 2	583	9:46	W, S, MS
Subject 1	1266	21:42	W, S, MS, RB
Subject 1	278	4:39	W, S, R
Subject 2	176	2:56	W, S, R
Subject 1	293	4:53	W, S, R
Subject 1	765	16:38	W, S, RB
Subject 2	1017	17:11	W, S
Subject 1	593	10:41	W, S, D
Subject 1	730	12:34	W, S, D

**Total**	**8498**	**2:46:01**	

**Table 2:** Distribution of the mobility classes.

**Mobility Class**	**No. of Instances**	**Percentage**
static	1144	13.5%
moving slowly	297	3.5%
walking	3443	40.5%
running	532	6.3%
driving a car	1135	13.4%
riding a bus	1482	17.4%
riding a train	465	5.5%

**Table 3:** Description of the features.

**Feature**	**Source**	**Description**
speed	GPS	Speed from GPS, converted to km/h.
speedChange	GPS	Difference in speed from previous GPS measurement.
accelVariance	accelerometer	Variance in the norm of the acceleration (Equation (4)) over the moving window.
headingChange	GPS	Sum of absolute value of heading changes over the moving window.
accuracy	GPS	Accuracy rating from GPS, assumed to be meters of horizontalerror.
trainDistance	GPS/GIS	Distance to the nearest train station in meters.
busDistance	GPS/GIS	Distance to the nearest bus stop in meters.
1 HzPeak	accelerometer	Strength of 10-Hz peak in FFT of the accelVariance signal.

**Table 4:** Comparison of algorithm performance.

**Classification Algorithm**	**Accuracy (% Correct)**	**Standard Deviation**
**Decision Trees**		
RandomForest	96.51	0.59
NBTree	95.40	0.71
J48graft	94.93	0.77
J48	94.83	0.81
RandomTree	94.45	0.93
LMT	93.25	1.29
FT	92.66	1.02
BFTree	93.77	0.83
REPTree	93.58	1.06
LADTree	85.37	1.29
SimpleCart	94.00	0.83

**Artificial Neural Networks**		
MultilayerPerceptron	87.15	1.80

**Bayesian Techniques**		
NaiveBayes	81.47	1.15
BayesNet	90.89	0.93

**Logistic Regression**		
Logistic	83.41	1.05

**Support Vector Machines**		
SMO	80.24	1.02

**Instance-Based Classifiers**		
KStar	95.55	0.70
IBk(kNN = 5)	80.32	1.25
LWL	95.45	0.14

**Baseline**		
ZeroR	40.52	0.06

**Table 5:** Feature selection analysis. LR, logistic regression; NB, naive Bayes.

**Feature set**	**RF**	**J48**	**MP**	**LR**	**NB**	**SV**
**Individual features**						

speed	72.9	73.8	71.4	67.7	71.5	55.5
accelVariance	64.2	70.7	62.2	57.4	55.6	57.3
trainDistance	61.6	69.0	56.1	51.7	53.9	50.7
speedChange	50.7	50.7	44.8	40.5	44.0	40.5
busDistance	49.7	56.9	45.6	43.4	43.3	42.9
accuracy	47.1	47.0	46.7	43.9	43.9	44.0
1HzPeak	46.3	51.6	49.8	45.0	43.6	40.5
headingChange	41.3	41.1	49.1	40.5	39.7	40.5

**All features, except**						

speedChange	97.7	95.9	90.1	87.1	79.7	85.0
headingChange	97.1	95.5	87.8	85.5	81.6	81.9
accelVariance	96.5	94.3	85.6	81.6	81.2	74.3
accuracy	96.2	94.3	85.9	83.6	79.5	78.9
speed	96.1	94.2	85.2	79.8	74.1	75.5
busDistance	96.1	94.8	86.5	82.7	82.0	78.7
1HzPeak (all time-domain features)	96.5	94.8	87.1	83.4	81.5	80.2
trainDistance	94.3	92.9	83.1	82.1	78.8	79.4

**All time-domain features, except**						

speedChange	96.7	94.9	86.9	83.5	81.4	80.2
headingChange	96.5	94.8	86.9	83.4	81.4	80.4
accelVariance	96.1	93.5	83.8	78.3	78.7	73.5
accuracy	95.6	93.6	85.1	81.8	78.0	77.6
busDistance	95.1	93.8	84.6	80.8	79.5	77.0
speed	94.6	92.1	83.0	77.6	72.9	73.1
trainDistance	93.3	92.0	82.4	81.1	76.7	77.8

**Other subsets**						

{av, s, t, b, acc, 1hp}	97.6	96.0	90.2	87.1	79.5	85.0
{av, s, t, b, acc}	96.5	94.9	86.6	83.5	81.2	80.2
{av, s, t, b, 1hp}	96.3	94.6	85.5	83.5	78.6	79.1
{av, t, b, acc, 1hp}	96.1	94.5	82.5	79.9	70.1	75.7
{av, s, t, b}	93.8	93.8	84.5	81.8	77.5	77.6
{av, s, h, acc, sc, hc, 1hp} (no GIS)	93.8	92.4	84.8	80.6	79.4	76.3
{av, s, t}	92.4	92.4	81.0	77.4	76.3	72.0
{b, t, 1hp}	89.6	88.4	67.4	62.3	55.2	57.4
{av, s, b}	89.4	89.4	80.8	79.0	73.4	75.0

**all**	**97.1**	**95.4**	**87.8**	**85.5**	**81.9**	**81.8**

**Table 6:** Comparison of computational time.

**Classification Algorithm**	**CPU Time (ms)**	**Standard Deviation**
**Decision Trees**		
RandomForest	7.96	7.84
NBTree	19.3	7.05
J48graft	3.74	6.70
J48	1.25	4.25
RandomTree	1.40	4.49
LMT	117	6.96
FT	762	54.5
BFTree	1.72	4.90
REPTree	1.09	4.00
LADTree	1.09	4.00
SimpleCart	1.25	4.25

**Artificial Neural Networks**		
MultilayerPerceptron	3.28	6.39

**Bayesian Techniques**		
NaiveBayes	13.26	6.79
BayesNet	6.55	8.05

**Logistic Regression**		
Logistic	4.21	6.96

**Support Vector Machines**		
SMO	2.65	5.90

**Instance-based Classifiers**		
KStar	7.21 × 10^4^	2.74 × 10^4^
IBk (kNN = 5)	751	16.9
LWL	8.60 × 10^5^	7.89 × 10^4^

**Table 7:** Confusion matrix for the RandomForest algorithm applied to the dataset.

		***Predicted Label***						

		**Walk**	**Static**	**Slow**	**Train**	**Bus**	**Run**	**Car**
*Actual Label*	**Walking**	**98.86**	0.572	0.145	0.000	0.032	0.383	0.006
	**Static**	3.024	**93.81**	1.792	0.079	0.420	0.236	0.638
	**Moving Slowly**	2.929	7.946	**87.85**	0.000	0.875	0.000	0.404
	**Riding Train**	0.000	0.323	0.000	**97.87**	1.742	0.022	0.043
	**Riding Bus**	0.297	0.499	0.115	0.803	**97.10**	0.007	1.174
	**Running**	6.184	0.959	0.000	0.019	0.019	**92.82**	0.000
	**Driving Car**	0.009	1.445	0.000	0.326	2.326	0.000	**95.89**
